# Pharmacokinetics and Bioequivalence of Two Formulations of Febuxostat 40-Mg and 80-Mg Tablets: A Randomized, Open-Label, 4-Way Crossover Study in Healthy Chinese Male Volunteers

**DOI:** 10.1371/journal.pone.0150661

**Published:** 2016-03-14

**Authors:** Zhu Luo, Feng Nan, Jia Miao, Zhihui Chen, Mei Li, Maozhi Liang

**Affiliations:** Institute of Drug Clinical Trials, West China Hospital, Sichuan University, Chengdu 610041, P. R. China; University of Bologna, ITALY

## Abstract

The present study aimed to investigate the pharmacokinetic properties of febuxostat in healthy Chinese male volunteers and evaluate whether the two formulations of febuxostat 40-mg and 80-mg tablets are bioequivalent. A randomized, open-label, 4-way crossover study was conducted in healthy Chinese male volunteers under fasting conditions. 24 eligible subjects were randomized in a 1:1:1:1 ratio to receive a single dose of test or reference formulation of febuxostat 40-mg or 80-mg tablet. The washout period between each administration was 1 week. Plasma febuxostat was quantified by a validated liquid chromatography-tandem mass spectrometry (LC-MS/MS) method. Tolerability was evaluated by monitoring adverse events, physical examinations, 12-lead ECG and laboratory tests. After single-dosing of 1 tablet of 40-mg febuxostat, the pharmacokinetic parameters of test and reference formulations were: T_max_ 1.22±0.87 and 1.85±1.03 h, C_max_ 1689.16±461.31 and 1613.80±608.43 ng·mL^-1^, AUC_0-t_ 5139.87±1349.28 and 5517.91±2024.26 ng·mL^-1^·h, AUC_0−∞_ 5263.06±1339.16 and 5640.48±2040.22 ng·mL^-1^·h, t_1/2_ 4.82±2.61 and 4.85±1.78 h, respectively. After single-dosing of 1 tablet of 80-mg febuxostat, the pharmacokinetic parameters of test and reference formulations were: T_max_ 1.71±1.21 and 2.23±1.55 h, C_max_ 2744.47±1157.44 and 2998.17±1200.13 ng·mL^-1^, AUC_0-t_ 9634.03±2768.25 and 10467.95±3501.65 ng·mL^-1^·h, AUC_0−∞_ 9834.32±2730.51 and 10626.63±3504.08 ng·mL^-1^·h, t_1/2_ 6.25±2.44 and 5.46±1.65 h, respectively. For single-dosing of 1 tablet of 40-mg febuxostat, 90% CIs for the test/reference ratio of AUC_0-t_, AUC_0−∞_ and C_max_ were 89.79 to 102.55, 90.14 to 102.56 and 93.99 to 129.63, respectively. For single-dosing of 1 tablet of 80-mg febuxostat, 90% CIs for the test/reference ratio of AUC_0-t_, AUC_0−∞_ and C_max_ were 86.67 to 100.00, 87.50 to 100.51 and 79.48 to 105.99, respectively. This single dose study revealed similar pharmacokinetic properties in healthy Chinese male volunteers as those found in Caucasic population. The test and reference febuxostat tablets formulations met the regulatory criteria for bioequivalence at 40-mg and 80-mg strengths in fasting healthy Chinese male volunteers.

***Trial Registration*:** Chictr.org ChiCTR-TTRCC-14004288

## Introduction

Gout is the most common inflammatory arthritis which affects nearly 2% of the total population.[1,2] The prevalence of gout has been increasing in many countries including the United States and China over the past decades.[3–5] Febuxostat is a novel xanthine oxidase (XO) inhibitor indicated for the chronic management of hyperuricemia in patients with gout. Many previous studies have indicated the efficacy and safety of febuxostat in lowering serum uric acid levels in patients with hyperuricemia and gout.[6–8]

The pharmacokinetic characteristics of febuxostat in human have been studied previously, including data available in Chinese population.[9–13] A previously published study compared the pharmacokinetics of febuxostat between Chinese and other races and indicated that some parameters in Chinese population appeared not to accord with the published literature in Caucasic population.[14]

Febuxostat was first developed in Japan and has been approved in many countries including the USA and the EU.[15,16] Recently, febuxostat was approved in China and recommended at 40 mg or 80 mg once daily. The dosage forms and strengths of the branded formulation in China are tablet 40-mg and 80-mg. Before allowing the marketing of a generic formulation, bioequivalence evaluation of the generic and branded formulations is required by China Food and Drug Administration (CFDA). The present study aimed to investigate the pharmacokinetic properties of febuxostat in healthy Chinese male volunteers and evaluate whether the two formulations of febuxostat 40-mg and 80-mg tablets are bioequivalent.

This was a registered study approved by China Food and Drug Administration. It was also registered in the World Health Organization International Clinical Trials Registry Platform (Chictr.org identifier: ChiCTR-TTRCC-14004288).

## Subjects and Methods

### Study design

**Fig 1:**
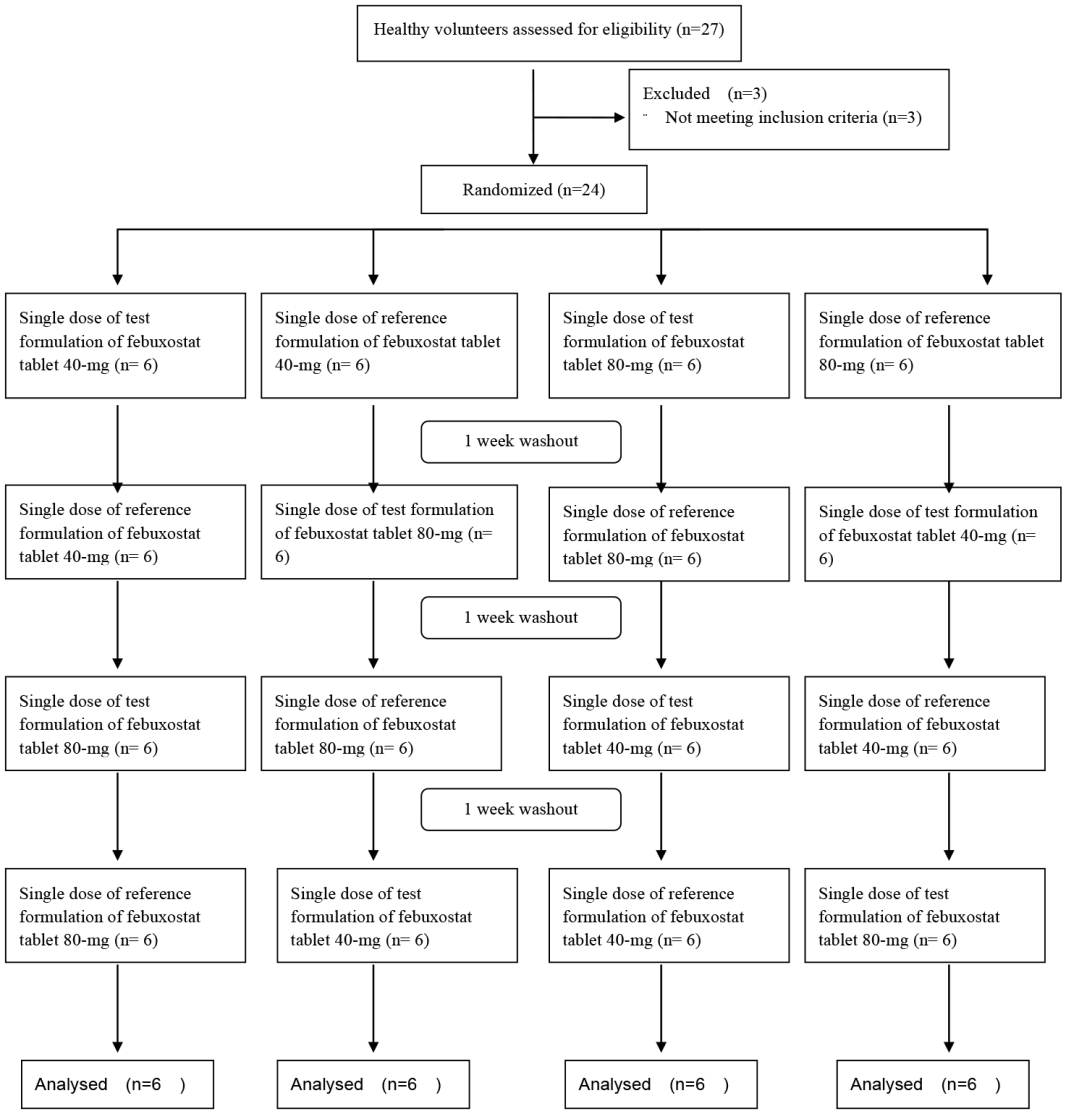
Flowchart of the study.

This was a randomized, open-label, 4-way crossover study planning to enroll 24 healthy Chinese male subjects. Based on a computer-generated table of random numbers, 24 subjects were allocated in a 1:1:1:1 ratio to receive a single dose of test or reference formulation of febuxostat 40-mg or 80-mg tablet under fasting conditions. The washout period between each administration was 1 week. The study flowchart is shown in Fig 1.

The study protocol was approved by the Independent Ethics Committee of West China Hospital, Sichuan University (Chengdu, China). The protocol for this trial and supporting CONSORT checklist are available as supporting information (see S1, S2 and S3 Files). The approval letter of ethic committee is available as supporting information S4 and S5 Files.

### Subjects

Healthy, nonsmoking Chinese male volunteers were recruited according to the following inclusion criteria: aged from 18 to 40, body mass index between 19–24 kg/m^2^, healthy status confirmed by physical examination, medical history, 12-lead ECG and laboratory tests (hematology, blood biochemistry, urinalysis, tests for alcohol and other drugs abuse). Exclusion criteria included any allergic history or history of cardiac, pulmonary, hepatic, renal, hematologic or gastrointestinal disease or any other acute or chronic disease. Written informed consent was obtained from each subject before screening procedures.

### Test and reference formulations

The generic febuxostat tablets (strengths: 40-mg and 80-mg; lot no. 131101S; expiration date Nov, 2015) manufactured by Beijing Furuikangzheng Pharmaceuticals Co. Ltd.(Beijing, People’s Republic of China) were used as test formulation. The branded febuxostat tablets (strengths: 40-mg and 80-mg; lot no. 1308720; expiration date Jan, 2015) manufactured by Jiangsu Wanbang Pharmaceuticals Co. Ltd.(Nanjing, People’s Republic of China) were used as reference formulation.

### Drug administration and sampling

The study drug administration and blood sampling were conducted in the Phase I Unit of West China Hospital, Sichuan University. In each period, the subjects were given a single dose of test or reference formulation of febuxostat 40-mg or 80-mg tablet after an overnight fast (12 hours). The febuxostat tablets were administered with 200 mL water. Additional water intake was permitted 2 hours after dosing. Standard meals (Food energy ~ 900 kcal; 30% protein, 60% carbohydrate, 10% fat) were offered 4 and 10 hours after dosing. Blood samples (~3.5 mL) were collected before and at 0.25, 0.5, 0.75, 1, 1.5, 2, 3, 4, 6, 8, 10, 12, 16, 24, 36, 48 hours after dosing. All the drug administration and blood sampling processes were under continuous medical supervision.

### Assays of febuxostat

**Fig 2:**
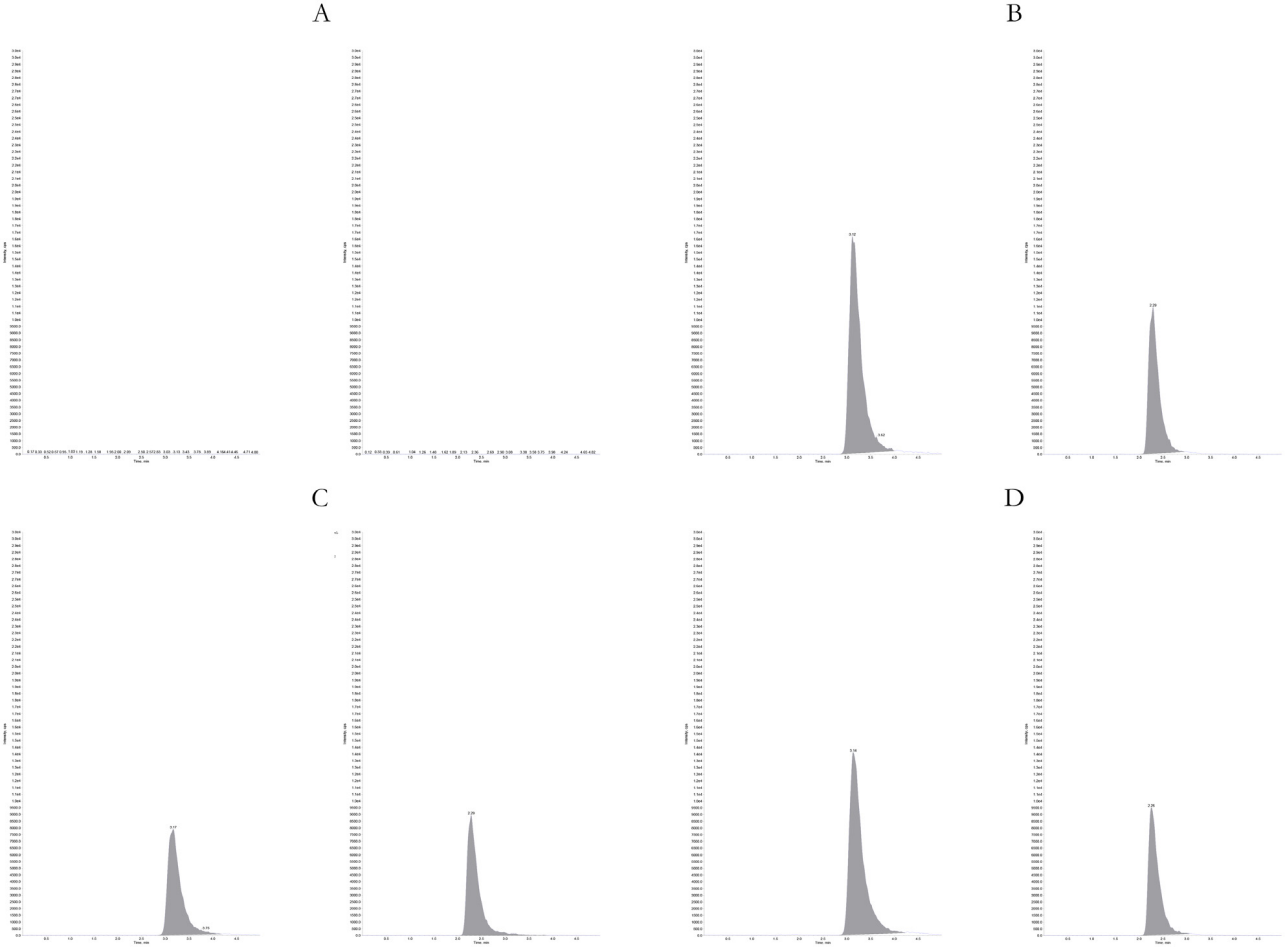
LC-MS/MS of febuxostat of blank plasma solution(A), reference standards solution (B), blank plasma with reference standards solution(C) and subject plasma solution (D).

Plasma febuxostat was quantified by a liquid chromatography-tandem mass spectrometry (LC-MS/MS) method developed and validated before the clinical study. Chromatography was performed using a Shimadzu SIL-HTC system (Shimadzu, Kyoto, Japan) equipped with a Ultimate C18 analysis column (50×4.6 mm, 5 μm). Mass spectrometric detection employed an API 3000 mass spectrometer with the working station Analyst 1.4.2 (AB Sciex, Ont., USA). The mobile phase consisted of acetonitrile-10mM ammonium acetate in water and formic acid (70:30:0.05, v/v/v) was delivered at a flow rate of 0.35 mL/min. Each 100 μL plasma sample was spiked with 100 μL internal standard (IS) solution. Then 100 μL hydrochloric acid (1mol·L^-1^) was added to acidize the spiked sample before the following extracting process. After extracted by 3.5 mL dichloromethane and then centrifuged, the supernatant was evaporated in a 45°C water bath. The residue was dissolved in 100 μL mobile phase and injected 10 μL onto the column. Bezafibrate was used as internal standard (IS). Negative multiple reaction monitoring (MRM) model was used and transitions were at m/z 315.1→271.1 and 361.9→275.6 for febuxostat and IS, respectively. The detailed MS parameters are shown in Table 1. Typical chromatograms are shown in Fig 2. The retention time for febuxostat and IS were 3.2 min and 2.3 min, respectively. The calibration curve was linear over the range of 10 ~ 4000 ng·mL^-1^. The lowest concentration of detection of febuxostat in plasma was 10 ng·mL^-1^. The method recovery was 98.6% ~ 100.4%. The intra-day RSD were less than 3% and inter-day RSD were less than 5%. The results of all stability studies were qualified for requirements.

**Table 1:** The mass spectrometry (MS) parameters for assays of febuxostat.

MS parameters	NEB	CUR	IS	TEM	DP	FP	EP	CE	CXP
febuxostat	6 V	6 V	5500 V	450°C	-23 V	-68 V	-8 V	-9 V	-6 V
bezafibrate	6 V	7 V	5500 V	450°C	-26 V	-96 V	-9 V	-24 V	-18 V

NEB = nebulizer gas, CUR = curtain gas, IS = ion spray voltage, TEM = source temperature, DP = declustering potential, FP = focus potential, EP = entrance potential, CE = collision energy, CXP = collision cell exit potential.

### Pharmacokinetics and bioequivalence analysis

WinNonlin Version 6.1 (Pharsight Corporation, Mountain View, California) was used to calculate the pharmacokinetic parameters of febuxostat with noncompartmental analysis method. C_max_ and T_max_ were obtained directly from the concentration–time data. Other pharmacokinetic parameters were calculated as previously reported[17]: AUC_0−t_ was calculated with the linear trapezoidal rule. AUC_0−∞_ was obtained as the sum of AUC_0−t_ and *Ct*/λ. *Ct* was the last measured concentration and λ was the slope of linear regression of the log-transformed concentration-time curve. t_1/2_ was calculated as 0.693/λ.

The relative bioavailability of the test formulation was calculated as AUC_0−t(test)_/AUC_0–t(reference)_ × 100%. 90% CIs for the test/reference ratio of log-transformed C_max_ and AUC were assessed by analysis of variance (ANOVA) using WinNonlin Version 6.1. Other pharmacokinetic parameters were analyzed using SPSS Version 18.0 (SPSS Inc. Chicago, IL, USA). T_max_ was tested by Wilcoxon signed rank test for significant differences. The two formulations were considered to be bioequivalent if the 90% CI for AUC was located within 80% to 125% and C_max_ within 70% to 143%, according to China Food and Drug Administration proposal.[18] The study’s sample size was estimated with Software PASS 11.0 (NCSS Statistical Software, Kaysville, Utah) based on the China Food and Drug Administration proposed bioequivalence range of 80% to 125% for AUC and 70% to 143% for Cmax, a power of 80% at an α of 0.05 to show bioequivalence. The test/reference mean ratio was estimated to be 0.95 to 1.05 and the within-subject CV was 25%.

### Tolerability assessment

Tolerability was evaluated by monitoring adverse events, physical examinations, laboratory tests (hematology, blood biochemistry and urinalysis) and 12-lead ECG. All the laboratory tests were performed in the laboratory of West China Hospital, Sichuan University and the laboratory was authenticated by College of American Pathologists (CAP).

## Results

### Subjects

Totally 24 male subjects were enrolled in 2 weeks and completed the study. The demographics of the subjects (mean ± SD, standard deviation) were: age 22.8 ± 1.4 (range, 21.0~26.0) years, weight 65.3 ± 7.5 (range, 50.0~76.0) kg, height 173.0 ± 5.0 (range, 160.0~179.0) cm, body mass index 21.8 ± 1.5 (range, 19.4~23.9) kg/m^2^.

### Pharmacokinetics of febuxostat

**Fig 3:**
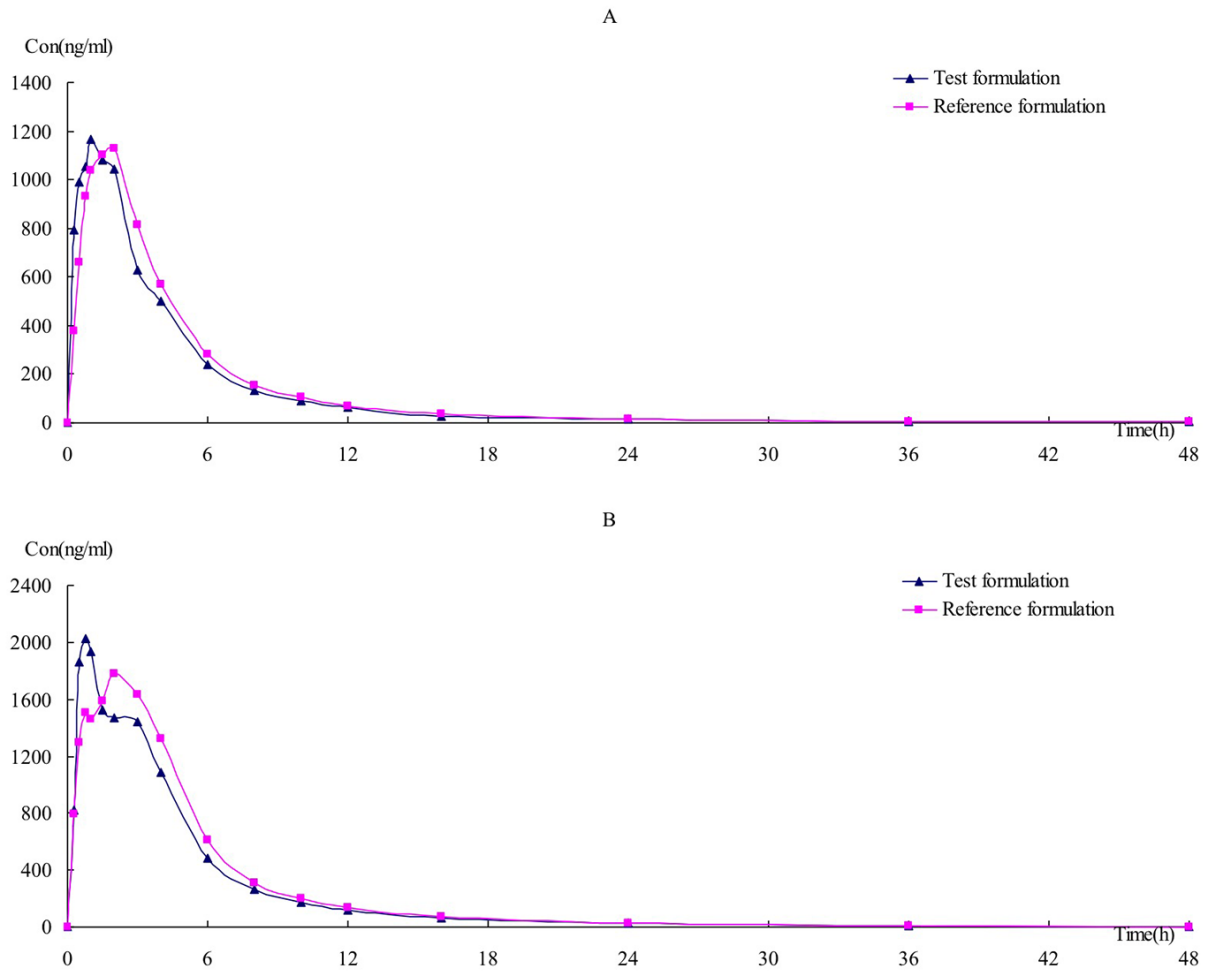
Mean plasma concentration-time curves of test and reference formulations of febuxostat after single-dosing of 1 tablet of 40-mg (A) and 80-mg (B).

Table 2 shows the pharmacokinetic parameters of febuxostat in 24 healthy Chinese male volunteers after single-dosing of 1 tablet of 40-mg or 80-mg febuxostat. The mean plasma concentration-time profiles of the two formulations after single-dosing of 1 tablet of 40-mg and 80-mg are shown in Fig 3.

**Table 2:** Pharmacokinetic parameters of febuxostat after single-dosing in healthy Chinese male volunteers.

PK parameter	40-mg		80-mg	
	test	reference	test	reference
T_max_, h	1.22±0.87	1.85±1.03	1.71±1.21	2.23±1.55
C_max_, ng•mL^-1^	1689.16±461.31	1613.8±608.43	2744.47±1157.44	2998.17±1200.13
AUC_0-t_, ng·mL^-1^·h	5139.87±1349.28	5517.91±2024.26	9634.03±2768.25	10467.95±3501.65
AUC_0−∞_, ng·mL^-1^·h	5263.06±1339.16	5640.48±2040.22	9834.32±2730.51	10626.63±3504.08
t_1/2_, h	4.82±2.61	4.85±1.78	6.25±2.44	5.46±1.65
MRT_0-t_, h	4.71±1.21	5.01±1.15	5.36±1.21	5.33±1.34
V/F, L	57.34±39.55	53.61±22.33	79.62±46.50	63.77±23.74
Cl/F, L/h	8.09±2.11	8.01±2.91	8.77±2.50	8.32±2.69

All values are mean±SD. (n = 24). Abbreviation: Tmax, time to reach maximum serum concentration; Cmax, maximum serum concentration; AUC, area under the serum concentration-time curve; t1/2, terminal half-life; MRT, mean residence time; V/F, apparent volume of distribution; Cl/F, clearance.

### Bioequivalence evaluation

Table 3 shows the 90% CIs for the test/reference ratio of log-transformed C_max_, AUC_0-t_, AUC_0−∞_. For single-dosing of 1 tablet of 40-mg febuxostat, 90% CIs for the test/reference ratio of AUC_0-t_, AUC_0−∞_ and C_max_ were 89.79 to 102.55, 90.14 to 102.56 and 93.99 to 129.63, respectively. For single-dosing of 1 tablet of 80-mg febuxostat, 90% CIs for the test/reference ratio of AUC_0-t_, AUC_0−∞_ and C_max_ were 86.67 to 100.00, 87.50 to 100.51 and 79.48 to 105.99, respectively. At both 40-mg and 80-mg strengths, the 90% CIs of AUC_0-t_ and AUC_0−∞_ were located within 80% to 125%, 90% CI for C_max_ was within 70% to 143%. The two formulations met the regulatory criteria for bioequivalence and were bioequivalent at 40-mg and 80-mg strengths.

**Table 3:** Bioequivalence evaluation of two formulations of febuxostat tablets after single-dosing in in healthy Chinese male volunteers.

	40-mg		80-mg	
	Test/Reference Ratio	90% CI	Test/Reference Ratio	90% CI
AUC_0-t_	95.96%	89.79~102.55	93.10%	86.67~100.00
AUC_0−∞_	96.15%	90.14~102.56	93.77%	87.50~100.51
C_max_	110.39%	93.99~129.63	91.78%	79.48~105.99

Data are shown as 90% CIs for the test/reference ratio of log-transformed C_max_, AUC_0-t_, AUC_0−∞_. (n = 24)

### Tolerability

Among the 24 subjects, only one reported abnormal laboratory tests (elevated transaminase) after administration, which was rated as mild and considered possibly associated with the study drug. No other Adverse Events were observed or reported. Single-dosing of febuxostat tablet at 40-mg and 80-mg strengths was considered to be well tolerated by healthy Chinese male volunteers.

## Discussion

Febuxostat is a novel xanthine oxidase (XO) inhibitor approved for the chronic management of hyperuricemia in patients with gout. The pharmacokinetic characteristics of febuxostat in human have been studied previously. After oral administration, febuxostat is well absorbed with the bioavailability about 84%. The time to reach the maximum plasma concentration is 1 to 1.5 hours and half-life is 5 to 8 hours. After a single of 40 mg and 80 mg of febuxostat, C_max_ is approximately 1.6 ± 0.6 μg/mL and 2.6 ± 1.7 μg/mL, respectively; AUC is approximately 4.0 ± 1.9 μg·h/mL and 9.1 ± 3.8 μg·h/mL, respectively.[11–13]

Pharmacokinetic data of febuxostat is also available in Chinese population. A previous study reported the pharmacokinetics of febuxostat in healthy Chinese volunteers and concluded the pharmacokinetics of febuxostat in Chinese appeared not in accord with the published literature in other races.[14] It was reported that C_max_ increased 27–58%, AUC increased 70–117%, half-life increased 20–57%, and CL/F decreased 44–57%, respectively, at 40–80 mg dose level for Chinese subjects. The author deduced that body weight and genetic variability should be taken into account to explain the observed ethnic difference.

It was reported that age or gender had no clinically significant effect on the pharmacokinetics of febuxostat.[13] Co-administration with food reduced the rate and extent of febuxostat absorption, however, this food effect on absorption was not associated with a clinically significant pharmacodynamic change.[12] Furthermore, febuxostat pharmacokinetic parameters for patients with hyperuricemia and gout estimated by population pharmacokinetic analyses were similar to those estimated in healthy subjects.[16,19] Considering the pharmacokinetic properties of febuxostat and the dosage forms and strengths of the branded formulation, we designed this randomized, open-label, 4-way crossover bioequivalence study in healthy male volunteers under fasting conditions.[20] Our study further investigated the pharmacokinetic properties of febuxostat in healthy Chinese male volunteers and evaluated the bioequivalence of two formulations of febuxostat 40-mg and 80-mg tablets. Our study indicated there were no significant differences between the pharmacokinetic parameters of the branded and generic formulations. The two formulations met the regulatory criteria for bioequivalence proposed by China Food and Drug Administration.

In the present study, we used a liquid chromatography-tandem mass spectrometry (LC-MS/MS) method to quantify plasma febuxostat. The method validation results indicated that the specificity, precision, accuracy, stability and recovery of the present method were comparable to those previously reported methods for the determination of febuxostat, suggesting our method was suitable for the determination of febuxostat in human plasma.[21,22]

In the present study, after a single dose of 40 mg and 80 mg of febuxostat tablets, the pharmacokinetic parameters accorded with those reported in Caucasic population.[11–13]

Compared with the subjects in the above mentioned study, the subjects in the present study were of more mean body weight and body mass index, which were more similar to the subjects of Caucasic population.[11–13] This might further confirm that the observed ethnic difference between Chinese and other races were caused by the subjects’ body weight. Another aspect to explain the metabolic difference is the genetic variability between subjects, however, we did not include polymorphism analysis in the present study, which is a limitation of this study. Another limitation of this study is that it only enrolled Chinese male volunteers and the data could not be extrapolated in female subjects or other races, which caused the lack of generalisability of the present study.

## Conclusions

The pharmacokinetic parameters in the present study accorded with those reported in Caucasic population and the test and reference febuxostat tablets formulations met the regulatory criteria for bioequivalence at 40-mg and 80-mg strengths in fasting healthy Chinese male volunteers.

## Supporting Information

S1 File CONSORT Checklist. (DOC)

S2 File English translation of study protocol abstract. (DOC)

S3 File Study protocol of febuxostat-Chinese version. (DOC)

S4 File English translation of EC approval letter. (DOC)

S5 File Approval letter of ethic committee. (PDF)
