# Supplementary material for: Pharmacokinetics and Bioequivalence of Two Formulations of Febuxostat 40-Mg and 80-Mg Tablets: A Randomized, Open-Label, 4-Way Crossover Study in Healthy Chinese Male Volunteers
Source: PLoS One. 2016 Mar 14;11(3):e0150661. doi: 10.1371/journal.pone.0150661 (PMC4790952; doi:10.1371/journal.pone.0150661)
Supplement: S2 File — (DOC) [file pone.0150661.s002.doc]

**Study title:** Pharmacokinetics and Bioequivalence of Two Formulations of Febuxostat 40-mg and 80-mg Tablets in Healthy Chinese Male Volunteers

**Study leader:** Zhu Luo

**Applicant's institution：**West China Hospital, Sichuan University

**Objectives of Study：**To study the Pharmacokinetics and Bioequivalence Febuxostat Tablets in Healthy Chinese male Volunteers.

**Study design****:** This was a randomized, open-label, 4-way crossover study planning to enroll 24 healthy Chinese male subjects. Based on a computer-generated table of random numbers, 24 subjects were allocated in a 1:1:1:1 ratio to receive a single dose of test or reference formulation of febuxostat 40-mg or 80-mg tablet under fasting conditions. The washout period between each administration was 1 week.

**Inclusion criteria:** Healthy male chinese volunteers aged from 18 to 40 and with a body mass index between 19–24 kg/m2; a healthy status confirmed by medical history, physical examination, 12-lead ECG, and laboratory tests (hematology, blood biochemistry, hepatic function, urinalysis, hepatitis B surface antigen, tests for alcohol and other drugs of abuse) and nonsmoking status.

**Exclusion criteria:** any allergic history or history of cardiac, pulmonary, renal, hepatic, gastrointestinal or hematologic abnormality or any other acute or chronic disease. exposure to any investigational medication within 30 days of the first dose of study medication, pregnant or nursing women, women of childbearing potential not using a highly effective method of birth control.

**Test and reference formulations**: The generic febuxostat tablets (strengths: 40-mg and 80-mg; lot no. 131101S; expiration date Nov, 2015) manufactured by Beijing Furuikangzheng Pharmaceuticals Co. Ltd.(Beijing, People’s Republic of China) were used as test formulation. The branded febuxostat tablets (strengths: 40-mg and 80-mg; lot no. 1308720; expiration date Jan, 2015) manufactured by Jiangsu Wanbang Pharmaceuticals Co. Ltd.(Nanjing, People’s Republic of China) were used as reference formulation.

**Drug administration and sampling**: The study drug administration and blood sampling were conducted in the Phase I Unit of West China Hospital, Sichuan University. In each period, the subjects were given a single dose of test or reference formulation of febuxostat 40-mg or 80-mg tablet after an overnight fast (12 hours). The febuxostat tablets were administered with 200 mL water. Additional water intake was permitted 2 hours after dosing. Standard meals (Food energy ~ 900 kcal; 30% protein, 60% carbohydrate, 10% fat) were offered 4 and 10 hours after dosing. Blood samples (~3.5 mL) were collected before and at 0.25, 0.5, 0.75, 1, 1.5, 2, 3, 4, 6, 8, 10, 12, 16, 24, 36, 48 hours after dosing. All the drug administration and blood sampling processes were under continuous medical supervision.

**Assays of febuxostat**: Plasma febuxostat was quantified by a liquid chromatography-tandem mass spectrometry (LC-MS/MS) method developed and validated before the clinical study. Chromatography was performed using a Shimadzu SIL-HTC system (Shimadzu, Kyoto, Japan) equipped with a Ultimate C18 analysis column (50×4.6 mm, 5 μm). Mass spectrometric detection employed an API 3000 mass spectrometer with the working station Analyst 1.4.2 (AB Sciex, Ont., USA). The mobile phase consisted of acetonitrile-10mM ammonium acetate in water and formic acid (70:30:0.05, v/v/v) was delivered at a flow rate of 0.35 mL/min. Each 100 μL plasma sample was spiked with 100 μL internal standard (IS) solution. Then 100 μL hydrochloric acid (1mol·L^-1^) was added to acidize the spiked sample before the following extracting process. After extracted by 3.5 mL dichloromethane and then centrifuged, the supernatant was evaporated in a 45 ℃ water bath. The residue was dissolved in 100 μL mobile phase and injected 10 μL onto the column. Bezafibrate was used as internal standard (IS). Negative multiple reaction monitoring (MRM) model was used and transitions were at m/z 315.1→271.1 and 361.9→275.6 for febuxostat and IS, respectively. The retention time for febuxostat and IS were 3.2 min and 2.3 min, respectively. The calibration curve was linear over the range of 10 ~ 4000 ng·mL^-1^. The lowest concentration of detection of febuxostat in plasma was 10 ng·mL^-1^. The method recovery was 98.6 % ~ 100.4 %. The intra-day RSD were less than 3% and inter-day RSD were less than 5%. The results of all stability studies were qualified for requirements.

**Pharmacokinetics and bioequivalence analysis**: WinNonlin Version 6.1 ( Pharsight Corporation, Mountain View, California ) is used to calculate the pharmacokinetic parameters of febuxostat with noncompartmental analysis method. C_max_ and T_max_ are obtained directly from the concentration–time data. AUC_0−t_ is calculated with the linear trapezoidal rule. AUC_0−∞_ is obtained as the sum of AUC_0−t_ and *Ct*/λ. *Ct* is the last measured concentration and λ is the slope of linear regression of the log-transformed concentration-time curve. t_1/2_ was calculated as 0.693/λ. The relative bioavailability of the test formulation is calculated as AUC_0−t(test)_/AUC_0–t(reference)_ × 100%. 90% CIs for the test/reference ratio of log-transformed C_max_ and AUC were assessed by analysis of variance (ANOVA) using WinNonlin Version 6.1. T_max_ was tested by paired Wilcoxon test for significant differences. The two formulations were considered to be bioequivalent if the 90% CI for AUC was located within 80% to 125% and C_max_ within 70% to 143%, according to China Food and Drug Administration proposal.

**Tolerability assessment**: Tolerability is evaluated by monitoring adverse events, physical examinations, laboratory tests (hematology, blood biochemistry and urinalysis) and 12-lead ECG. All the laboratory tests are performed in the laboratory of West China Hospital, Sichuan University and the laboratory was authenticated by College of American Pathologists (CAP).
