# Supplementary material for: Pharmacokinetics and Bioequivalence of Two Formulations of Febuxostat 40-Mg and 80-Mg Tablets: A Randomized, Open-Label, 4-Way Crossover Study in Healthy Chinese Male Volunteers
Source: PLoS One. 2016 Mar 14;11(3):e0150661. doi: 10.1371/journal.pone.0150661 (PMC4790952; doi:10.1371/journal.pone.0150661)
Supplement: S3 File — (DOC) [file pone.0150661.s003.doc]

**SFDA临床试验批件号：2011L00222，2010L04996 化学药品注册分类3.1类**

**非布司他片人体生物等效性试验**

**试验方案**

**密级: 机密**

申办单位：北京福瑞康正医药技术研究所

临床试验单位：四川大学华西医院国家药物临床试验机构

主要研究者：罗柱 南峰

方案撰写：罗柱

方案版本号：第1版 2013.08.30

**签 名 页**

**研究单位：四川大学华西医院国家药物临床试验机构**

地址：四川省成都市国学巷37号

邮编：610041

机构负责人： 梁茂植 签名：

试验负责人： 罗 柱 签名：

联系电话： 028-85422709

E-mail: luozhu720@163.com

方案拟定人： 罗 柱 签名：

方案审核人： 梁茂植

原始资料保存地：四川大学华西医院国家药物临床试验机构

**申办单位：北京福瑞康正医药技术研究所**

地址：北京市丰台区靛厂795号

邮编：100039

项目负责人： 顾群 签名：

监查员：刘海龙 签名：

联系电话：18612362335 010-88400085-815

E-mail: hailong.liu@bj-jyyy.com

# 目录

[目录 1](#__RefHeading___Toc301426647)

[研究者和研究管理结构 4](#__RefHeading___Toc301426648)

[方案修改记录 5](#__RefHeading___Toc301426649)

[试验方案同意书 6](#__RefHeading___Toc301426650)

[方案摘要 7](#__RefHeading___Toc301426651)

[缩略语 9](#__RefHeading___Toc301426652)

[1. 背景资料 10](#__RefHeading___Toc301426653)

[1.1 药物简介](#__RefHeading___Toc301426654) 11

[1.2临床前主要药效学研究资料](#__RefHeading___Toc301426655) 11

[1.3临床前毒理学研究资料](#__RefHeading___Toc301426656) 11

1.4 国外临床研究资料 11

[2. 试验目的 10](#__RefHeading___Toc301426657)

[3. 试验方法 12](#__RefHeading___Toc301426658)

[3.1 试验设计 12](#__RefHeading___Toc301426659)

[3.2 试验流程 12](#__RefHeading___Toc301426660)

[3.3 试验对象 13](#__RefHeading___Toc301426661)

[3.4受试者替代 14](#__RefHeading___Toc301426666)

[3.5受试者试验禁忌和合并用药 14](#__RefHeading___Toc301426667)

[3.6 受试者饮食注意事项 14](#__RefHeading___Toc301426668)

[3.7 受试者筛选 14](#__RefHeading___Toc301426669)

[3.8 受试者随机化 14](#__RefHeading___Toc301426670)

[3.9受试者评估 15](#__RefHeading___Toc301426671)

[4 试验药物 15](#__RefHeading___Toc301426674)

[4.1 试验制剂与参比制剂 15](#__RefHeading___Toc301426675)

[4.2试验制剂的保管和发放 15](#__RefHeading___Toc301426676)

[4.3试验制剂包装和标签 15](#__RefHeading___Toc301426677)

[4.4 试验药物给药 15](#__RefHeading___Toc301426678)

[5.临床观察及抢救措施 16](#__RefHeading___Toc301426679)

[6.安全性评价 16](#__RefHeading___Toc301426680)

[7.不良事件 16](#__RefHeading___Toc301426681)

[7.1预期的不良事件 16](#__RefHeading___Toc301426682)

[7.2 不良事件的观察和记录 16](#__RefHeading___Toc301426683)

[7.3 严重不良事件 17](#__RefHeading___Toc301426684)

[7.4 异常实验室检查值随访 18](#__RefHeading___Toc301426685)

[8.样品采集 18](#__RefHeading___Toc301426688)

[8.1设计依据 18](#__RefHeading___Toc301426689)

[8.2 样品采集 19](#__RefHeading___Toc301426695)

[8.3 进餐与采血 19](#__RefHeading___Toc301426698)

[9. 分析样品 19](#__RefHeading___Toc301426699)

[9.1 样品标签 19](#__RefHeading___Toc301426700)

[9.2 样品保存和转运 20](#__RefHeading___Toc301426701)

[10. 样品分析 20](#__RefHeading___Toc301426702)

[10.1 仪器及色谱条件 20](#__RefHeading___Toc301426703)

[10.2 化学对照品 20](#__RefHeading___Toc301426704)

[10.3 分析方法评价标准 20](#__RefHeading___Toc301426705)

[10.4 样品分析随行质控 21](#__RefHeading___Toc301426706)

[11. 数据处理与分析 21](#__RefHeading___Toc301426707)

[11.1 样品检测 21](#__RefHeading___Toc301426708)

[11.2 血药浓度及药-时曲线 21](#__RefHeading___Toc301426709)

[11.2 药动学参数 21](#__RefHeading___Toc301426710)

[11.3 生物等效性评价 21](#__RefHeading___Toc301426711)

[11.4 统计处理方案 21](#__RefHeading___Toc301426712)

[12. 伦理和管理 21](#__RefHeading___Toc301426713)

[12.1 独立的医学伦理委员会 22](#__RefHeading___Toc301426714)

[12.2 知情同意和知情同意书 22](#__RefHeading___Toc301426715)

[12.3主要研究者职责 22](#__RefHeading___Toc301426716)

[12.4申办方职责 22](#__RefHeading___Toc301426717)

[12.5 监查员职责 22](#__RefHeading___Toc301426718)

[13. 试验质量控制 23](#__RefHeading___Toc301426719)

[14. 试验报告提供资料 23](#__RefHeading___Toc301426720)

[15. 试验文件、病例报告表及记录的保存 23](#__RefHeading___Toc301426721)

[15.1 原始文件/数据 23](#__RefHeading___Toc301426722)

[15.2病例报告表 24](#__RefHeading___Toc301426723)

[15.3 研究者档案/文件保存 24](#__RefHeading___Toc301426724)

[16. 试验文件/受试者记录的保密 24](#__RefHeading___Toc301426725)

[17. 资料发表和商业秘密保护 24](#__RefHeading___Toc301426726)

[18. 试验进度 25](#__RefHeading___Toc301426727)

[19. 附件：试验流程图 26](#__RefHeading___Toc301426727)

# 研究者和研究管理结构

本试验由四川大学华西医院国家药物临床试验机构负责，对非布司他片进行人体生物等效性试验。试验单位及其职责见下表。

| 单位 | 研究者或联系人 | 职责 |
| --- | --- | --- |
| 北京福瑞康正医药技术研究所 | 顾 群 | 申办者 |
|  |  |  |
| 北京福瑞康正医药技术研究所 | 刘海龙 | 临床监查 |
|  |  |  |
| 四川大学华西医院国家药物临床试验机构I期病房 | 罗 柱 | 受试者筛查、药品管理、给药、临床观察，CRF表填写，血浆样品采集药动学、统计学和生物等效性分析、研究报告撰写 |
| 四川大学华西医院国家药物临床试验机构I期临床试验研究室 | 南 峰 | 血浆非布司他检测方法的建立，方法学验证，血浆样品测定、 |
|  |  |  |
| 四川大学华西国家药物临床研究机构 | 梁茂植 | 流程和质量管理，原始资料保存 |

# 方案修改记录

| 修改日期 | 修改内容 | 是否报伦理备案 | 研究者签名 |
| --- | --- | --- | --- |
|  |  |  |  |
|  |  |  |  |
|  |  |  |  |

# 试验方案同意书

以下研究者和申办者代表的签字表明他们均认可此临床试验方案，并确保该临床试验按照GCP原则和方案中的相关规定进行，其中包括保密条例。双方均同意就这一试验的执行过程和结果进行保密。该试验的病例报告表和其它数据属北京福瑞康正医药技术研究所所有，试验原始资料保存在四川大学华西医院国家药物临床试验机构。双方均同意这一试验方案中包含了执行这一试验的所有必要信息。另外该试验的执行必须得到四川大学华西医院医学伦理委员会批准。双方均同意所有受试者需提供书面的知情同意书，以符合GCP原则和赫尔辛基宣言的要求。所有受试者的研究记录将被保密(国家和四川省食品药品监督管理局稽查除外)。

机构负责人：梁茂植 签名： 日期：

临床负责人：罗 柱 签名： 日期：

实验负责人：南 峰 签名： 日期：

申办方负责人：顾 群 签名： 日期：

监查员：刘海龙 签名： 日期：

# 方 案 摘 要

| **申办方** | 北京福瑞康正医药技术研究所 |
| --- | --- |
| **试验机构** | 四川大学华西医院国家药物临床试验机构 |
| **项目负责人** | 罗柱 |
| **试验题目** | 非布司他片人体生物等效性试验 |
| **试验目的** | 通过健康志愿受试者分别单次口服由北京福瑞康正医药技术研究所开发的试验制剂非布司他片与市售参比制剂非布司他片，进行非布司他片相对生物利用度研究，评价试验制剂与参比制剂的生物等效性。 |
| **试验设计** | 本试验为两阶段、开放、随机、交叉、四周期、口服给药的单中心试验。经体检合格男性健康志愿受试者，按1：1：1：1比例随机分组，分别交叉口服由40mg和80mg两个阶段试验北京福瑞康正医药技术研究所开发的非布司他片与参比制剂非布司他片，评价试验制剂40mg与80mg两个规格片剂与参比制剂的生物等效性。清洗期设计为7天。 |
| **受试人数** | 24例符合纳入标准的健康男性受试者。 |
| **试验制剂** | 北京福瑞康正医药技术研究所提供非布司他片，规格：40mg和80mg，批号：131101S ，有效期：24个月 。 |
| **参比制剂** | 江苏万邦生化医药股份有限公司生产的非布司他片，规格：40mg， 批号：1308720有效期：2015年1月31日 。 |
| **给药剂量** | 试验制剂(非布司他片)或参比制剂非布司他片(商品名：优立通 ) ，40mg或80mg规格1片。 |
| **给药方法** | 受试者空腹用温水200 mL口服。 |
| **样本采集** | 每周期给药前空白点及给药后0.25、0.5h、0.75、1h、1.5h、2h、3h、4h、6h、8h、10h、12h、16h、24h、36h、48h，每一时间点取静脉血3.5mL，共17次(含空白血样，共计60mL)。采集的血样置肝素抗凝试管，立即离心分离血浆，置-40°C冰箱避光储存。 |
| **分析方法** | 采用HPLC-MS/MS法测定非布司他血药浓度，内标法定量。 |
| **药动学研究** | 采用Phoenix™ WinNonlin® 6.1程序，拟合非布司他的经时血药浓度数据。主要药动学参数AUC_0-t_、AUC_0-∞_、Cl、Vd、t_1/2_按统计矩计算，T_max_、C_max_用实测值。 |
| **生物等效性评价** | 以各个受试者非布司他片试验制剂(A)和参比制剂(R)的AUC_0-t_按下式分别计算其相对生物利用度(F)值：F=AUC_A_/AUC_R_×100%。经对数转换后的试验制剂的AUC_0-t_、AUC_0-∞_在参比制剂的80%-125%范围，Cmax在参比制剂的70%-143%范围，根据双单侧检验的统计量，同时求得(1-2α)%置信区间，如在规定范围内，即可由(1-2α)的概率判断两药生物等效。Tmax采用非参数法秩和检验统计，如无差异，可以认定试验制剂与参比制剂生物等效。 |
| **统计分析** | 试验数据均用均数和标准差及相对标准差表示，药物动力学参数的计算用Phoenix™ WinNonlin® 6.1程序进行。AUC_0-t_，AUC_0-∞_，Cmax经对数转换后进行多因素方差分析，假设检验界值默认为α=0.05，采用双单侧*t*检验及90%置信区间法判断单次口服受试制剂与参比制剂的生物等效性。Tmax经非参数秩和检验进行分析。其他药动学参数使用SPSS统计学软件进行配对样本t检验，*P*值小于或等于0.05被认为所检验的差别有统计意义。 |
| **安全性评价** | 口服非布司他片试验制剂或参比制剂至少一次的受试者都将被纳入安全性资料的评价中。不良事件将记录在病历记录表中并在研究结束后进行总结。安全性评价指标包括受试者生命体征的变化，实验室检查值的变化，并计算不良事件发生率。 |
| **试验周期** | 本试验计划在2013年10月开始。临床部分需要8周，样品测定、数据处理、统计分析、资料总结和报告撰写大约需要12周，共20周。 |

# 缩 略 语

| **缩写** | **中文全称** | **英文全称** |
| --- | --- | --- |
| A | 白蛋白 | Albumin |
| AE | 不良事件 | Adverse Event |
| ALT | 丙氨酸氨基转移酶 | Alanine Aminotransferase |
| AST | 天冬氨酸氨基转移酶 | Aspartate Transaminase |
| AUC_(0-t)_ | 从0到t时间药-时曲线下面积 | Area Under The Curve From Zero To t Time Point |
| AUC_(0-∞)_ | 时间外推到无限药–时曲线下面积 | Area under the curve from zero to infinite |
| BUN | 尿素氮 | Blood urea nitrogen |
| Cl | 药物清除率 | Clearance of drug |
| C_max_ | 血药峰浓度 | Maximum plasma concentration |
| Cr | 肌酐 | Creatinine |
| CRF | 病例报告表 | Case report form |
| DB | 直接胆红素 | Direct bilirubin |
| G | 球蛋白 | Globulin |
| GCP | 药物临床试验质量管理规范 | Good clinical practice |
| Hb | 血红蛋白 | Hemoglobin |
| Mean | 均数 | / |
| MRT_(0-t)_ | 从0到t时间的平均滞留时间 | Mean retention time from zero to t time point |
| MRT_(0-∞)_ | 时间外推到无限的平均滞留时间 | Mean retention time from zero to infinite |
| ND | 未检出 | Not detected |
| PLT | 血小板计数 | Platelet count |
| SAE | 严重不良事件 | Serous adverse event |
| SD | 标准差 | Standard deviation |
| SFDA | 国家食品药品监督管理局 | State food and drug administration |
| SOP | 标准操作规程 | Standard operating procedure |
| T_max_ | 达到峰值血药浓度的时间 | Time to maximum plasma concentration |
| t_1/2_ | 消除半衰期 | Elimination half-life |
| TB | 总胆红素 | Total bilirubin |
| TP | 总蛋白 | Total protein |
| Vd | 表观分布容积 | Volume of distribution |
| WBC | 白细胞计数 | White blood cell count |

**非布司他片人体生物等效性试验**

**试验方案**

由北京福瑞康正医药技术研究所开发的第3.1类化学药品非布司他片已获国家药品监督管理局临床批件，批件号为2011L00222，2010L04996。该品种以3.1类申报，但在临床批件审批期间，国内已有同品种获得生产批件。故虽然本品种分类上仍属3.1类，但本研究拟进行本品与国内已上市的同品种生物等效试验。

按《化学药物制剂人体生物利用度和生物等效性试验技术指导原则》要求，通过试验评价试验制剂北京福瑞康正医药技术研究所开发的化学药品非布司他片与参比制剂(江苏万邦生化医药股份有限公司生产的非布司他片)相对生物利用度及是否具有生物等效性。

## 1. 背景资料

**1.1药物简介**

非布司他是2-芳基噻唑衍生物，通过选择性抑制XO来降低血清尿酸而达到疗效。2008年4月欧盟批准非布司他片上市，规格为80mg、120mg，商品名为Adenuric®，用于治疗尿酸盐沉积的高尿酸血症，上市公司为Beaufour Ipsen Pharm。2009年2月13日，美国FDA批准非布司他片上市，用于有高尿酸血症的痛风患者的慢性治疗。规格为40mg、80mg，商品名为ULORIC，上市公司为武田制药北美公司。

**1.2 临床前主要药效学研究资料**

非布司他抑制牛乳中黄嘌呤氧化酶及小鼠和大鼠肝脏中黄嘌呤氧化酶/黄嘌呤脱氢酶的lC_50_分别为1.4、1.8和2.0nmol/L，而别嘌呤醇的相应lC_50_分别为l700、380和1100nmol/L。非布司他对牛乳中黄嘌呤氧化酶所产生的抑制作用为混合型(Ki=0.7nmol/L)，而别嘌呤醇的抑制作用则表现为竞争型(Ki=280 nmol/L)。

与服用别嘌呤醇相比，给正常大鼠和小鼠口服非布司他降低血中尿酸浓度的作用更强(小鼠服药后2小时的ED_50_为0.7 *vs*2.7 mg/kg)，且作用持续时间更长。重复给予正常大鼠口服非布司他(1-100mg/kg，每日1次，28天)增加血液及尿液中黄嘌呤浓度的作用较服用别嘌呤醇(3-200mg/kg，每日1次)强10~30倍，而在黄嘌呤排泄率及肾结石形成方面两种药物无差别。本品用于尿囊噁酸钾(potassium oxonate，250mg/kg，sc，给予本品前1小时)所致高尿酸血症大鼠同样具有疗效。

**1.3 临床前毒理学研究资料**

**1.3.1 急性毒性**

非布司他对大鼠的近似致死剂量为300～600mg/kg p.o.（口服）。最高非致死剂量为300mg/kg p.o.（雄鼠+雌鼠)。给药后6小时～4天出现死亡。患病动物的临床表现：5-25分后自发运动减少、紫绀、侧卧或俯卧、体温降低。尸检：胃有溃疡样黑色斑点、回肠和十二指肠变色（溢血）。

比格犬口服2000mg/kg非布司他，未观察到死亡。最高非致死剂量为2000mg/kg p.o.。临床表现为严重呕吐（所有剂量组都出现）、俯卧、侧卧、消沉、自发运动减少、上睑下垂、体表温度下降、流涎、稀便。给药1天后观察到临床表现全部开始恢复。

**1.3.2 长期毒性**

研究非布司他对小鼠、大鼠、犬、黑猩猩口服给药的长期毒性。试验显示主要靶器官为肾、膀胱、甲状腺、肝和造血系统。

对比格犬的12个月毒性研究表明15mg/kg非布司他（约为人血浆暴露量的35倍，80mg/天）导致犬肾内沉积黄嘌呤结晶。大鼠6个月的毒性研究也出现相似的结果，48mg/kg非布司他（约为人血浆暴露量的35倍，80mg/天）导致大鼠肾内沉积黄嘌呤结晶。

致突变性：一系列致突变试验显示非布司他没有任何生物学相关的基因毒性。

致癌性：雄性大鼠服用高剂量（约人体暴露量的11倍）非布司他，患膀胱肿瘤（移性细胞乳头状瘤和癌）的机率明显增多，认为与黄嘌呤结石有关。雄性或雌性小鼠、大鼠患其他类型肿瘤的机率没有显著增加。这些发现被认为与物种特异性的嘌呤代谢和尿形成有关，与临床使用没有关系。

生殖毒性：雄性和雌性大鼠口服48 mg/kg/天非布司他，药物对生育力和生殖行为没有影响。没有证据显示非布司他会损伤生育力，有致畸作用或对胎儿有害。高剂量（约为人体暴露量的4.3倍）的非布司他有母体毒性，导致大鼠离乳指数降低，子代的发育减慢。对妊娠大鼠（约为人体暴露量的4.3倍）和妊娠兔子（约为人体暴露量的13倍）进行致畸性试验表明非布司他无致畸作用。

**1.4 国外临床研究资料**

2项3期关键性试验证实了ADENURIC的疗效，1832例高尿酸血症和痛风患者参与。在每个3期关键性试验中，ADENURIC降低并维持血清尿酸（sUA）水平均优于别嘌呤醇。主要疗效指标是最后3个月均测得血清尿酸水平﹤6.0mg/dl(357μmol/l)的患者比例。这些试验不包含器官移植患者。

APEX试验：是一项3期、随机、双盲、多中心、28周的试验，别嘌呤醇和安慰剂为对照。1072例患者随机分为：安慰剂组（n=134），ADENURIC 80mg QD（1天4次）组(n=267)，ADENURIC 120 mg QD组(n=269)，ADENURIC 240 mg QD组(n=134)或别嘌呤醇300 mg QD组（患者的基线血清肌酐≤1.5 mg/dl [n=258]），别嘌呤醇100 mg QD组（患者的基线血清肌酐﹥1.5mg/dl，且≤2.0 mg/dl，[n=10]）。240mg非布司他（推荐剂量的2倍）作为安全性评估剂量。试验表明ADENURIC 80 mg QD和ADENURIC 120 mg QD组的疗效显著优于别嘌呤醇300 mg（n=258）/100 mg（n=10）组，将sUA降低至6.0 mg/dl(357μmol/l)以下。

FACT试验：是一项3期、随机、双盲、多中心、52周的试验，别嘌呤醇为对照。760例患者随机分为ADENURIC 80 mg QD组(n=256)，ADENURIC 120 mg QD组(n=251)，或别嘌呤醇300 mg QD组(n=253)。试验表明ADENURIC 80 mg QD和ADENURIC 120 mg QD的疗效显著优于别嘌呤醇300 mg，将sUA降低并维持至6.0 mg/dl(357μmol/l)以下。

## 2. 试验目的

通过健康志愿受试者分别单次口服由北京福瑞康正医药技术研究所开发的试验制剂非布司他片与市售参比制剂非布司他片(商品名：优立通)，分别进行非布司他相对生物利用度研究，评价试验制剂与参比制剂的生物等效性。

## 3. 试验方法

### 3.1 试验设计

本试验为两阶段、开放、随机、四周期、交叉、单中心试验。经体检合格男性健康志愿受试者，按1：1：1：1比例随机分组，分别交叉口服由40mg和80mg两个阶段试验北京福瑞康正医药技术研究所开发的非布司他片与参比制剂非布司他片，评价试验制剂40mg与80mg两个规格片剂与参比制剂的生物等效性。清洗期设计为7天。

### 3.2 试验流程

(1) 研究人员及申办方遵照GCP原则及有关规定，查阅文献资料，共同拟定试验方案、CRF及知情同意书。

(2) 试验相关资料报四川大学华西医院医学伦理委会审查批准。

(3) 受试者24例，先后参加40mg和80mg两个阶段试验，每阶段随机分为两组，分别交叉口服由北京福瑞康正医药技术研究所开发的非布司他片与参比制剂非布司他片。

(4) 样品药物浓度测定。

(5) 数据处理和分析。

(6) 资料整理。

(7) 撰写研究报告。

### 3.3 试验对象

本试验计划从志愿者中纳入24例男性健康受试者，按照《化学药物制剂人体生物利用度和生物等效性试验技术指导原则》(2005年3月)及《药品注册管理办法》，进行受试者筛选。

### 3.3.1 纳入标准

(1) 男性，年龄18~40岁。

(2) 受试者体重指数在19~24之间。

(3) 健康体检，包括体格检查、病史、生命体征、心电图和临床实验室检查（血、尿常规，肝肾功，乙肝标志物，HIV抗体）合格。

(4) 自愿受试并签署知情同意书。

### 3.3.2 排除标准

(1) 体检及实验室检查超正常范围且研究者认为具有临床意义者。

(2) 过敏体质，有药物过敏史、或/和过敏性疾患者。

(3) 有嗜烟、酗酒史或药物滥用史者。

(4) 有急、慢性消化道疾病和心、肝、肾等重要器官疾病史者；有糖尿病，甲状腺功能亢进，帕金森综合征，或者已知能够干扰试验结果的任何其它疾病或生理情况。

(5) 有血液系统疾病失或有明显出血倾向者。

(6) 有慢性精神疾患或精神异常者。

(7) 试验前2周内，使用过任何其他药物(包括中药)者。

(8) 在参加本次试验前30天内参加过其它药物试验。

(9) 在开始试验前3月内有严重的失血或捐献血液或血浆。

### 3.3.3中止标准

(1) 试验中出现不能耐受的不良反应者。

(2) 不依从试验方案，或自动要求退出试验者。

(3) 研究者认为受试者有安全性可能受到损害的危险。

### 3.3.4 退出标准

受试者有权在任何时间以任何理由退出试验。研究者在下面情况下有权让受试者退出试验，如并发症、不良事件和因违反方案服药或其它原因。但是可以理解的是，退出率过高会导致试验无法解释；因此，研究者应避免不必要的退出。如果一个受试者决定退出，那么应尽可能详尽地完成和报告所有观察结果。受试者退出时应做一个完整的最终评估，同时需写明退出原因。

如果受试者因为不良事件或实验室检查结果异常退出试验，则需在病例报告表中记录相关重要细节。

### 3.4受试者替代

纳入足够的受试者，尽量确保无需受试者替代。

### 3.5受试者试验禁忌和合并用药

在试验开始前3天和整个试验期间禁止服用酒精及含酒精的饮料。试验开始前24小时和整个试验期间禁止饮用任何含咖啡因的饮料(如茶、咖啡、可口可乐、可乐等)。

在试验期间禁止吸烟。第一次服药前24小时和整个试验期间禁止剧烈活动。

试验期间禁止服用任何非试验药品。如果受试者服用了任何药物，必须通知研究者，并将详细情况记录在病例报告表中。

### 3.6 受试者饮食注意事项

受试者在各阶段采血当日只能接受由试验单位提供的标准的不含咖啡因的食物和饮料。试验期间所有受试者统一进食标准餐。两个试验阶段的用餐和用餐时间相同，以保证两个试验阶段的一致性。

### 3.7 受试者筛选

试验方案经四川大学华西医院医学伦理委员会批准后，将从志愿者中选出受试者。首先，他们将给予口头和书面的关于本试验的说明，并给予充足时间让他们考虑是否希望参加。若他们决定参加本试验，将签署知情同意书，在试验开始前进行筛选。研究者将对受试者进行包括一般资料(年龄、身高、体重)，病史和体格检查，测量心率和血压，取尿液进行常规检查，取血进行生化、血液学、乙肝和HIV等检查，并进行心电图检查。受试者必须符合所有的纳入标准且不符合排除标准。研究者将保留所有受试者的筛选记录，同时应记录受试者未被纳入原因。任何近期的用药情况也将详细记录。

### 3.8 受试者随机化

按计算机生成的随机数字表，在每个试验阶段将24名受试者随机分为两组，每组12人。

### 3.9受试者评估

### 3.9.1 依从性评估

试验期间研究者应确保受试者对试验过程的依从性。任何违背方案的情况都需要研究者和申办者进行评估，以确定受试者是否继续试验。

### 3.9.2可评价受试者的标准

受试者完全符合入选标准并至少服用了一次药物，即使中途因故退出试验也需作为安全性评价病例。

## 4 试验药物

### 4.1 试验制剂与参比制剂

试验制剂：非布司他片，由北京福瑞康正医药技术研究所生产提供，规格：每片含非布司他40mg和每片含非布司他80mg，批号：131101S ，有效期：24个月 。

参比制剂：非布司他片，由江苏万邦生化医药股份有限公司生产，规格：每片含非布司他40mg，批号：1308720有效期：2015年1月31日。

### 4.2试验制剂的保管和发放

试验药物应在符合《药品生产质量管理规范》条件的车间制备，并经检验符合质量标准。申办单位将试验药物发放到试验单位，有发放和签收记录。试验单位有专人保管试验药物，记录药物使用情况。试验药物分配表应随时更新并包括以下内容：(1) 受试者编号；(2) 发放给受试者的药物数量和日期。试验期间申办单位监查员可随时检查药物管理记录。试验结束后剩余药物和使用药物应与记录相符。研究人员应保存全部的药物管理记录。试验结束时剩余药物由监查员收回或由研究者交回至北京福瑞康正医药技术研究所。北京福瑞康正医药技术研究所提供的所有试验药物只能用于本次人体生物等效性试验，不能用于其它目的。

### 4.3试验制剂包装和标签

所有试验药物的包装均应符合 GCP的要求。标签应注明试验编号、药物名称、用药方法和数量、批号、失效期。每名受试者试验药物为一个小包装，包装标签包含：项目代码、受试者随机号、试验周期、试验组别、试验日期。药物放置在规定的存放地点，同时包装易于拆开。

### 4.4 试验药物给药

由指定的研究者为受试者发药，受试者试验当日清晨空腹用温开水200mL口服非布司他片试验制剂或参比制剂，药物应和水一起整粒吞服，不应咀嚼或压碎。试验药物在研究者监督下服用。

## 5.临床观察及抢救措施

四川大学华西医院I期临床试验病房具有各级医护人员组成的医疗监护小组，密切观察可能出现的不良反应；试验现场有受试者休息场所和病床，备有心电图仪、除颤器、呼吸机、洗胃机、静脉切开包、气管插管、氧气袋等抢救设施和急救药品，以确保受试者用药后的医学监护及安全。I期病房有过敏、低血压等不良反应处理预案。对出现严重不良反应者应立即终止试验，并采取有效的急救措施。

## 6.安全性评价

安全性评价指标包括受试者主述、症状、生命体征(体温、脉搏、呼吸、收缩压、舒张压)和体格检查的变化；实验室检查值的变化：红细胞计数、血红蛋白、血小板计数、白细胞计数、总胆红素、直接胆红素、丙氨酸转氨酶、天冬氨酸转氨酶、尿素氮、肌酐、尿常规和心电图等，并计算不良反应发生率。

## 7.不良事件

### 7.1预期的不良事件

根据国内外文献记载的非布司他临床试验中发生的不良反应，非布司他片人体生物等效性试验预期的不良反应可能有：肝功能测试结果异常、便秘、腹泻、胃食管返流、恶心、呕吐、腹部不适、头痛、关节相关的症状和体征（关节痛、关节肿胀）、肌肉骨骼和结缔组织的症状和体征（背部、胸壁、腰窝或手足疼痛和肌肉骨骼僵硬）、眩晕、皮疹、外周水肿等。大部分的不良事件都是轻度和中度的。

### 7.2 不良事件的观察和记录

研究者应严格按照GCP规定如实记录和处理不良事件，记录内容包括不良事件的表现、发生时间、严重程度、持续时间、处理的措施和转归。

所有在试验期间发生的不良事件，无论受试者在试验期间自发报告的，还是由研究者引述的均应记录在病例报告表中。不良事件是指服药受试者发生的任何不良的医疗事件，但是与本试验不一定有必然的因果关系。因此不良事件可以是任何一种不良的未预见的体征(包括异常实验室化验值)、症状或在时间上与使用药物有关的疾病，无论实际是否与试验药物有关。此外，不良事件也可以指受试者任何基线症状(给药前)的未预见的变化(包括身体、心率或行为改变)，包括给药开始后发生的间发性疾病，不管它是否与试验药物有关。“给药”包括在试验期间使用的所有试验药物。与正常的生长发育有关其发生率和程度没有变化的不作为不良事件。

过量给药是指故意或非故意给予高于试验方案规定和高于已知治疗剂量的药物。不论什么结果都必须报告，即使没有观察到毒副反应。

按照下列标准判断不良事件的程度，并详细填写病例报告表：

轻度：容易耐受，只引起很小的不适，不影响正常生活。

中度：足以影响正常生活的不适。

重度：致残和/或妨碍正常生活。

不良事件与试验药物的关系按肯定有关、很可能有关、可能有关、可能无关、无关五级进行，并在CRF中填入代号。肯定有关、很可能有关、可能有关纳入不良反应发生率计算。

对任何在随访时仍继续发展的与试验药物或试验方法有关的不良事件将继续进行随访，直到不良事件解决并且研究者在受试者最后一次复查后再随访两周。如果不能很快解决，试验单位确定的任何解决方案必须记录在试验档案中并复印作为研究者的记录。如果在试验总结报告完成之前，不良事件未得到解决，提交报告时应附上关于解决此不良事件的详细情况。

### 7.3 严重不良事件

包括异常化验值在内的任何临床不良事件，符合下面严重不良事件定义，而且发生在试验期间，不论受试者是否接受了治疗，必须在研究者得知情况的24小时内电话报告给四川大学华西医院医学伦理委员会和北京福瑞康正医药技术研究所。随后在严重不良时间表中详细记录下列内容：发生时间，痊愈时间，发生频率，严重程度，针对试验药物所采取的措施，采取的治疗，与试验药物的关系，此事件是否严重以及到目前的结果如何。电话报告后应书面报告与不良事件有关的详细情况。

严重不良事件是指可导致下列任何一种情况的不良事件：死亡；危及生命(根据初始报告者的看法，一旦发生即可导致受试者立即死亡的不良事件，它不包括那些如果更严重些可能导致死亡的不良事件)；永久性或明显伤残(伤残指能导致受试者正常生活能力永久性的伤害)；须住院治疗或延长住院时间；先天畸形/出生缺陷。没有导致死亡、危及生命，或住院的严重医疗事件，但经过恰当的医疗鉴定后，被认为可能危害受试者，并且需要内科/外科治疗才能阻止上述严重不良事件定义中的情况出现时，也可以看作是严重不良事件。

在试验期间出现的或在停止治疗4周内研究者获知的死亡，不论是否与治疗有关都必须报告。

如受试者在临床试验中出现任何严重不良事件，无论此事件是否与临床试验及试验用药物有关，研究者应立即对事件做出全面的评价并完成严重不良事件表，在24小时内报告有关省、自治区、直辖市(食品)药品监督管理部门和国家食品药品监督管理局及申办者，并及时向伦理委员会汇报。出现严重不良事件者将被中止试验。严重不良事件随访至恢复正常后15日，或直到得到妥善解决或病情稳定。

四川大学华西医院医学伦理委员会 联系电话：028-85422654

国家食品药品监督管理局安监司 联系电话：010-68313344-1013

申办方项目负责人：顾群 刘海龙 联系电话：010-88400085

主要研究者： 罗柱 联系电话：028-85421606

### 7.4 异常实验室检查值随访

### 7.4.1 筛选期

试验方案经四川大学华西医院医学伦理委员会批准后，将从志愿受试者中筛选本试验受试者。首先，由研究者向他们介绍本临床试验相关内容，同时他们将阅读关于本试验的“受试者须知”，如果了解了本试验相关内容后他们希望参加试验，研究者将给予充分的时间考虑，如果他们决定参加本临床试验，将签署知情同意书，在试验开始前15天内，依据方案的入选标准和排除标准筛选合适的健康受试者进入本试验。

如果在筛选期出现异常的实验室检查值，应立即重新检查。研究者将审查此结果并判断是否具有临床意义。如果此结果有临床意义，则受试者不能纳入试验。如果研究者认为必要，可以继续随访受试者。

受试者必须符合所有纳入标准且不符合排除标准方可进行本试验。研究者将保留所有受试者筛选记录，同时应记录受试者未被纳入的原因，任何近期的用药情况也将详细记录。筛选合格的受试者将给予一个试验编号。

### 7.4.2 随访期

如果在随访期出现异常的实验室检查值，研究者将首先判断有无临床意义；对有临床意义的实验室异常需随访至结果恢复正常或基线水平，该类实验室异常应当作为不良事件记录在病例报告表中，并详细记录处理和转归。

## 8.样品采集

### 8.1设计依据

### 8.1.1 剂量设计依据

根据本品规格及临床用法用量，计划评价的剂量为40mg和80mg。

### 8.1.2 参比制剂设计依据

目前国内已上市的非布司他片由江苏万邦生化医药股份有限公司生产，故选择该制剂作为参比制剂。

### 8.1.3 受试者例数设计依据

按照《化学药物制剂人体生物利用度和生物等效性试验技术指导原则》(2005年3月)，非布司他片人体生物等效性试验计划纳入24例男性受试者，用于(1) 初步考察非布司他片试验制剂与参比制剂的药动学特点，有无生物等效性；(2) 初步评价非布司他片的安全性；(3) 考察分析方法的灵敏度能否达到本试验的检测要求，确定标准曲线线性范围。

### 8.1.4 给药方法设计依据

为减少食物对生物等效性结果的影响，给药方法为：受试者试验当日清晨空腹用温开水200mL口服非布司他片试验制剂或参比制剂，药物应和水一起整粒吞服，不应咀嚼或压碎。服药2 h后可饮水，4 h后进标准餐。

### 8.1.5 样品采集设计依据

根据已有研究，口服非布司他片后1~2小时达到血浆峰浓度，半衰期为5~6小时。按照《化学药物制剂人体生物利用度和生物等效性试验技术指导原则》(2005年3月)，考虑非布司他吸收相、分布相和消除相各时段内均有取样点，达峰前至少取3个点，达峰时间取1~2个点，达峰后取6或以上时间点，整个采样时间应包括5~7个半衰期，故确定本试验采血时间为服药前和服药后0.25、0.5h、0.75、1h、1.5h、2h、3h、4h、6h、8h、10h、12h、16h、24h、36h、48h，每一时间点取静脉血3.5mL，共17次(含空白血样，共计60mL)。清洗期设计为7天，以保证在下一阶段给药时99.99%以上的药物已从体内消除。

### 8.2 样品采集

受试者于试验前日晚进食低脂清淡饮食并入住I期试验病房，次日晨于一侧前臂静脉安置留置针(可保留至给药后12小时)，抽取空白血样3.5mL，两组受试者分别空腹用200mL温开水口服非布司他片试验制剂与参比制剂，并于给药后0.25、0.5h、0.75、1h、1.5h、2h、3h、4h、6h、8h、10h、12h、16h、24h、36h、48h，每一时间点取静脉血3.5mL，共17次(含空白血样，共计60mL)，置肝素抗凝试管，立即离心分离血浆，置-40°C冰箱避光储存待测。

### 8.3 进餐与采血

进餐时间与采血时间冲突时，采血应在用餐前完成。

## 9. 分析样品

### 9.1 样品标签

每个样品管采用打印标签，标签包括以下内容：

(1) 项目代码

(2) 受试者代码

(3) 试验周期和组别

(4) 样品编号和时间。

在病例报告表上明确标明受试者采样日期和受试者编号，同时病例报告表必须有项目代码、受试者编号以及试验周期和组别。

### 9.2 样品保存和转运

本试验在四川大学华西医院国家药物临床试验机构的I期病房和药物分析研究室进行，不存在样品转运，样品一经采集即进行分离处理，直接于药物分析研究室指定冰箱保存。

## 10. 样品分析

### 10.1 仪器及色谱条件

采用HPLC-MS/MS法检测，仪器包括岛津公司SIL-HTC系列HPLC仪与AB公司API 3000三重四级杆串联质谱仪。图谱的获取和分析处理均由AB公司Analyst色谱工作站完成。

### 10.2 化学对照品

非布司他对照品与内标对照品均购自中国食品药品检定研究院，纯度为供含量测定用。

### 10.3 分析方法评价标准

HPLC-MS/MS法测定人血浆中非布司他浓度的分析进行方法学验证，包括：

(1) 生物样品中内源性物质不干扰样品色谱峰；

(2) 标准曲线的线性范围应包括全部研究样品的浓度范围，对浓度大于标准曲线上限的样品，按稀释系数用相同空白基质稀释后进行测定；

(3) 样品携带污染率，小于10%；

(4) 最低定量限应能满足测定5~7个半衰期时样品中的药物浓度，或 Cmax 1/10~1/20时的药物浓度；

(5) 高、中、低方法学验证样品的批内、批间相对标准差(RSD)小于15%；在LLOQ附近的RSD小于20%；

(6) 高、中、低方法学验证样品及内标的绝对回收率大于50%且稳定；

(7) 高、中、低方法学验证样品及内标的基质效应在±15%间且稳定；

(8) 高、中、低方法学验证样品的方法回收率应在85%~115%范围；

(9) 考察非布司他血浆样品室温放置、-40°C条件下长期保存、-40°C条件下反复冻融的稳定性；同时考察预处理好后的分析样品室温放置，预处理好后的分析样品复溶物在进样室中放置和重复进样的稳定性。要求与0时刻比较，浓度变化率应在±15%之间。

### 10.4 样品分析随行质控

按样品测定方法操作，每天为1个分析批次，配制标准曲线并同时测定高、中、低3个浓度的质控样品，每个浓度至少双样本，并应均匀分布在未知样品测试顺序中。要求质控样品的准确度在85%~115%范围，LLOQ附近质控样品的准确度在80%~120%范围，最多允许1/3的质控样品结果超限，但不能出现在同一浓度质控样品中。根据质控样品的测定浓度是否在方法误差的允许范围内，判定本次测定结果是否有效。若质控样品测定结果判定为不在控，则本批次样品应重新处理后测定。

## 11. 数据处理与分析

### 11.1血药浓度及药-时曲线

测定受试者单次口服非布司他片试验制剂和参比制剂后各时间点的血药浓度数据，并绘制药-时曲线。

### 11.2 药动学参数

采用Phoenix™ WinNonlin® 6.1程序，拟合非布司他的经时血药浓度数据。主要药动学参数AUC_0-t_、AUC_0-∞_、Cl、Vd、t_1/2_按统计矩计算，T_max_、C_max_用实测值。

### 11.3 生物等效性评价

以各个受试者非布司他片试验制剂(A)和参比制剂(R)的AUC_0-t_按下式分别计算其相对生物利用度(F)值：F=AUC_A_/AUC_R_×100%。经对数转换后的试验制剂的AUC_0-t_、AUC_0-∞_在参比制剂的80%-125%范围，Cmax在参比制剂的70%-143%范围，根据双单侧检验的统计量，同时求得(1-2α)%置信区间，如在规定范围内，即可由(1-2α)的概率判断两药生物等效。Tmax采用非参数法秩和检验统计，如无差异，可以认定试验制剂与参比制剂生物等效。

### 11.4 统计处理方案

试验数据均用均数和标准差及相对标准差表示，药物动力学参数的计算用Phoenix™ WinNonlin® 6.1程序进行。AUC_0-t_，AUC_0-∞_，Cmax经对数转换后进行多因素方差分析，假设检验界值默认为α=0.05，采用双单侧*t*检验及90%置信区间法判断单次口服受试制剂与参比制剂的生物等效性。Tmax经非参数秩和检验进行分析。其他药动学参数使用SPSS统计学软件进行配对样本t检验，*P*值小于或等于0.05被认为所检验的差别有统计意义。

## 12. 伦理和管理

### 12.1 独立的医学伦理委员会

本临床试验方案(包括任何改动)及知情同意书须得到四川大学华西医院医学伦理委员会的批准。

### 12.2 知情同意和知情同意书

在向受试者充分解释本试验试验目的、方法和可能的药物不良反应之后，研究者有责任获得每个受试者的书面知情同意书。研究者必须向受试者解释清楚他们有权力拒绝参加试验或随时退出试验。

影响到研究者的方案改动或最新的试验药物安全性资料(研究者手册)将反映到知情同意书中，而且受试者需重新签名。试验当中，如果因为试验药物使受试者致病或造成伤害，受试者将接受及时必要的医疗救治，并按照当地法规给予相应经济补偿，费用和责任由申办单位承担。

### 12.3主要研究者职责

主要研究者确保本临床试验完全符合国家法律、条例规定和赫尔辛基宣言。主要研究者负责准备报伦理委员会审批资料：试验方案、知情同意书、病例报告表、伦理申请表。主要研究者确保试验按照试验方案进行，并及时真实填写病例报告表，完成各类试验记录分析总结。四川大学华西医院国家药物临床试验机构I期病房负责完成受试者的筛选、体检、培训、服药、采血、血浆分离、医学监护等工作。四川大学华西医院I期临床试验药物分析研究室负责试验方案和知情同意书的拟定及试验的组织实施，建立非布司他血浆药物浓度HPLC-MS/MS测定方法并进行方法学评价，完成本试验受试者血浆样品药物浓度检测、数据处理、总结资料撰写等工作。

### 12.4申办方职责

提供试验所需要的研究者手册、各类临床前和临床资料，及时向研究者提供试验相关医学、药学和技术信息。

### 12.5 监查员职责

北京福瑞康正医药技术研究所的临床研究监查员有责任定期联系并拜访研究者。在符合对受试者保密的条件下，允许他/她对各方面试验记录(病例报告表和其他相关资料)进行检查。临床试验监查员的责任是在整个试验期间定期检查病例报告表，以确保试验单位对方案的遵守，表格填写的完整性、一致性和准确性。临床试验监查员必须核实每一受试者对试验记录和试验过程都有书面知情同意书。研究者将配合临床试验监查员，以确保在监查过程中发现的问题得以解决。临床试验监查员在完成每次检查后应撰写监查报告，分别存档于申办单位和临床试验单位。

## 13. 试验质量控制

**13.1** 四川大学华西医院国家药品临床试验机构I期病房及I期临床试验药物分析研究室的设施和条件符合国家食品药品监督管理局规定进行药物人体药动学和生物利用度试验的要求，本试验的所有主要研究人员全部参加了“国家新药临床试验GCP培训”并取得资格证书，具有承担该项临床试验的专业特长、资格和能力。试验开始前对研究者(包括护理人员)进行试验方案的培训。

**13.2** 四川大学华西医院国家药物临床试验机构I期病房有受试者筛选、体检、培训、服药、采血、血浆分离、医学监护等相关的标准操作规程和质量控制程序。

**13.3** 四川大学华西医院I期临床试验药物分析研究室有方法学建立及评价、样品测定及保存、数据质量保证等相关的标准操作规程和质量控制程序。由质量主管指定非本项目组人员作项目质控，按照本试验机构内部质控程序执行质控。

**13.4**临床质控员和实验室质控员按照机构相关质控程序全程质控，填写指控记录和质控报告，对抽查不吻合的数据和有疑问的数据将填入质控报告中提交研究者解决，研究者查证原始资料及相关信息书面回答质控提出的问题，更正需由研究者确认签字。当数据被确认为完整和准确后进行锁定和备份。

所有记录的更改修正均需有研究者签名和更改日期。

## 14. 试验报告提供资料

(1) 受试者分别单次口服非布司他片试验制剂和参比制剂后，各例受试者及其平均血药浓度-时间数据。

(2) 受试者分别单次口服非布司他片试验制剂和参比制剂后，各例受试者及其平均血药浓度-时间曲线。

(3) 受试者分别单次口服非布司他片试验制剂和参比制剂后，各例受试者及其平均药动学参数。

(4) 受试者分别单次口服非布司他片试验制剂和参比制剂统计学分析结果。

(5) 非布司他片试验制剂与参比制剂生物等效性分析结果。

(6) 受试者的人口学资料、体检和实验室检查结果、不良反应等资料。

(7) 研究结论。

## 15. 试验文件、病例报告表及记录的保存

### 15.1 原始文件/数据

将能够成为临床试验原始资料的文件和病例报告表中的原始数据资料在试验开始前归入研究者内部档案。监查过程中需与原始资料核实的病例报告表中的数据也应被看作原始数据，并归入研究者内部档案。

人体生物等效性试验试验数据被确认为完整和准确后进行电子版双备份；打印方法学及样品测定色谱图，并打印药动学、生物等效性和统计分析计算原始过程。

### 15.2病例报告表

对于凡用过一次试验用药物的每一位受试者，研究者必须完成一份病例报告表并在其上签名，并保证每份病例报告表中的数据正确和完整。如果受试者因为不良事件退出临床试验，研究者须尽可能记录试验结果。受试者评估过程中或评价结束之后，研究者应立即填写病例报告表，表格填写字迹必须清楚。如填写错误，可在错误处划一单线，而不应该涂抹或用修正液覆盖，同时填入修正内容，研究者需在修正处签名并注明日期。

临床试验监查员在监查过程中将检查病例报告表和原始病历、原始记录等的一致性，发现的病例报告表中的错误和与原始资料不吻合需声明并改正。所有更改之处需主要研究者核实确认签字并签更改日期，所有病例报告表归档保存。

### 15.3 研究者档案/文件保存

研究者必须按照当地规定或政府部门的要求保留以上文件资料和记录、受试者一般资料至研究结束或停止后至少5年，超过5年时间研究者可与北京福瑞康正医药技术研究所协商解决。如有特殊问题和/或法律要求，研究者在保证受试者相关资料保密的情况下，需提供完整的研究记录。

## 16. 试验文件/受试者记录的保密

研究者必须为受试者保密。交至申办方的病例报告表和其他文件，受试者只需凭编号确定，而不提供姓名。

需单独建立一个受试者档案，内容包括受试者编号、姓名和地址。不需交至申办方的文件，由研究机构严格保密保存。

## 17. 资料发表和商业秘密保护

经与申办方协商同意，研究者可将临床试验的信息或结果发表在任何科学杂志或其他出版物上，也能用于教学和其他科研活动。

由申办者提供给研究者的资料(包括本临床试验方案)均属非公开信息，必须保密。未经申办单位和临床试验机构负责人书面许可，任何人都不得以任何方式将资料泄露给他人。对违反本规定的单位和个人，将追究其责任并按有关规定处罚。

## 18. 试验进度

(1) 2013年11月底前完成受试者生物样品采集，分析方法的建立及验证。

(2) 2014年1月底前完成血药浓度测定。

(4) 2014年2月底前完成数据处理及分析和研究报告。

**非布司他片人体生物等效性试验**

**流程图**

| **项目** | **筛选**  **基线期** | **第一阶段（40mg）** | | | **清洗期** | **第二阶段（80mg）** | | | **试验**  **结束** |
| --- | --- | --- | --- | --- | --- | --- | --- | --- | --- |
|  |  | **第一周期** | **清洗期**  **1周** | **第二周期** | **1周** | **第一周期** | **清洗期**  **1周** | **第二周期** |  |
|  | **-14~0d** |  |  |  |  |  |  |  |  |
| 知情同意书 | **X** |  |  |  |  |  |  |  |  |
| **人口学资料和病史** | **X** |  |  |  |  |  |  |  |  |
| **吸烟史、饮酒史** | **X** |  |  |  |  |  |  |  |  |
| **入选/排除标准** | **X** |  |  |  |  |  |  |  |  |
| **体格检查** | **X** |  |  |  |  |  |  |  | **X** |
| **血液常规** | **X** |  |  |  |  |  |  |  | **X** |
| **尿常规** | **X** |  |  |  |  |  |  |  | **X** |
| **血生化** | **X** |  |  |  |  |  |  |  | **X** |
| **肝炎标志物** | **X** |  |  |  |  |  |  |  |  |
| **ECG** | **X** |  |  |  |  |  |  |  | **X** |
| **服药** |  | **X** |  | **X** |  | **X** |  | **X** |  |
| **临床监护** |  | **X** |  | **X** |  | **X** |  | **X** |  |
| 血药浓度采集 |  | **X** |  | **X** |  | **X** |  | **X** |  |
| **合并用药记录** | **X** | **X** | **X** | **X** | **X** | **X** | **X** | **X** | **X** |
| **不良事件记录** |  | **X** | **X** | **X** | **X** | **X** | **X** | **X** | **X** |
