# Supplementary material for: Pharmacokinetics and Bioequivalence of Two Formulations of Febuxostat 40-Mg and 80-Mg Tablets: A Randomized, Open-Label, 4-Way Crossover Study in Healthy Chinese Male Volunteers
Source: PLoS One. 2016 Mar 14;11(3):e0150661. doi: 10.1371/journal.pone.0150661 (PMC4790952; doi:10.1371/journal.pone.0150661)
Supplement: S4 File — (DOC) [file pone.0150661.s004.doc]

Approval Certificate of Independent Ethics Committee of West China Hospital, Sichuan University

NO: 2013, clinical trial (51)

| Department: Institute of Drug Clinical Trials | PI: Zhu Luo, M.D. |
| --- | --- |
| Study drug: febuxostat | Dosage form: tablets (40-mg/80-mg) |
| NDA to China Food and Drug Administration | Registration type: 3.1 |
| Registered number of CFDA: 2010L04996 | |
| Sponsor: Beijing Furuikangzheng Pharmaceuticals Co. Ltd.(Beijing, People’s Republic of China) | |
| Study title: Pharmacokinetics and Bioequivalence of Two Formulations of Febuxostat 40-mg and 80-mg Tablets in Healthy Chinese Male Volunteers | |
| EC approval type: By committee discuss | |
| EC discuss site: Room 413, Number 8 Medical Teaching Building of West China Hospital, Sichuan University | |
| EC review opinions:  1. The PI meets the qualification requirements of EC.  2. The study protocol and Informed Consent Form meet the requirements of EC and GCP guideline.  Result of EC review: Approved | |
