# Supplementary material for: Pharmacokinetics and Bioequivalence of Two Formulations of Febuxostat 40-Mg and 80-Mg Tablets: A Randomized, Open-Label, 4-Way Crossover Study in Healthy Chinese Male Volunteers
Source: PLoS One. 2016 Mar 14;11(3):e0150661. doi: 10.1371/journal.pone.0150661 (PMC4790952; doi:10.1371/journal.pone.0150661)
Supplement: S5 File — (PDF) [file pone.0150661.s005.pdf]

四川大学华西医院临床试验与生物医学伦理专委会审查批件

2013年临床试验（西药）审（ 51 ）号

科室（专业）：GCP中心	负责人/职称：罗柱　医师
药物名称：非布司他片	剂型：片剂<br>规格：80mg
向SFDA的申请事项：新药	注册分类：化药3.1类
药物临床研究批件号：SFDA“药物临床试验批件”：2010L04996	
申办单位：北京福瑞康正医药技术研究所	
研究项目名称：非布司他片人体生物等效性试验	
审查方式：　■会议审查　　□快速审查	
审查会议地点：四川大学华西医院老八教413会议室	

审评意见：

1. 研究者资质符合伦理要求。
2. 研究方案及知情同意书基本符合伦理要求。

审查结果：■同意　□作必要修正后同意　□作必要修正后再审　□不同意　□终止或暂停

持续审查频率：□3 个月/3months　□6 个月/6months　■1 年/1year　□不适用/NA

请遵循我国相关法律、法规和规章（SFDA《药物临床试验质量管理规范》（2003）、《医疗器械临床试验规定》（2004）、WMA《赫尔辛基宣言》和CIOMS《人体生物医学研究国际道德指南》、卫生部《涉及人的生物医学研究伦理审查办法（试行）（2007）》），遵循伦理委员会批准的方案和知情同意书开展临床试验（研究），保护受试者的健康与权利。

在试验（研究）过程中，若变更主要研究者，对临床研究方案、知情同意书等的任何修改，请申请人提交修正案审查申请。

发生严重不良事件，请申请人及时提交严重不良事件报告；紧急报告之后，尽快提交详细的严重不良事件随访报告。

请递交年度和定期跟踪审查报告；当出现任何可能显著影响试验（研究）进行或增加受试者危险的情况时，请申请人及时向伦理专委会提交书面报告。

试验（研究）纳入了不符合纳入标准或符合排除标准的受试者，符合中止试验（研究）规定而未让受试者退出试验（研究），给予错误治疗或剂量，给予方案禁止的合并用药等没有遵从方案开展研究的情况；或可能对受试者的权益/健康、以及研究的科学性造成不良影响等违背伦理原则与规范的情况，请申办者/监查员/研究者提交违背方案报告。

申请人暂停或提前终止临床试验（研究），请及时提交暂停/终止试验（研究）报告。

完成临床试验（研究），请申请人提交结题报告。

本批件有效期为一年，逾期未实施的，则自行废止。

单　位（章）：四川大学华西医院 5101070022002

主任委员（签名）：[signature]

日期：2013 年 9 月 13 日

联系方式：成都市武侯区国学巷 37 号四川大学华西医院老八教 412，孙荣国/左泽锦/李娜　　电话/传真：028-85422654

（附件见背面）

附件：

伦理审查文件

1.方案（版本：第1版，日期：2013.08.30）

2.知情同意书

3.临床试验批件

4.药检报告

5.病例报告表

6.研究者手册

四川大学华西医院临床试验与生物医学伦理专委会会议参会委员名单

姓名	性别	专业	职称	工作单位	签字	日期
曾勇（主任委员）	男	肝胆胰外科	教授	四川大学华西医院	[illegible]	2013.9.10
孙荣国	男	医学管理	教授	四川大学华西医院	[illegible]	2013.9.10
毛兵	男	中西医结合科	教授	四川大学华西医院	毛兵	2013.9.10
张瑞明	女	中西医结合科	教授	四川大学华西医院	[illegible]	2013.9.10
冯萍	女	传染科	教授	四川大学华西医院	冯萍	2013.9.10
朱焕玲	女	血液科	教授	四川大学华西医院	朱焕玲	2013.9.10
兰礼吉	男	伦理学	教授	四川大学政治学院	兰礼吉	2013.9.10
傅政勇	男	法律学	律师	中豪律师集团（四川）事务所	傅政勇	2013.9.10
赵建芳	女	教育学	教师	成都市武侯计算机实验小学	赵建芳	2013.9.10
